# Box Lesion Isolation of the Left Atrial Posterior Wall with Radiofrequency Ablation Restricted in Predetermined Lines for the Treatment of Persistent Atrial Fibrillation: The Prognostic Role of Acute Interventional Outcome and Trigger Identification

**DOI:** 10.19102/icrm.2023.14115

**Published:** 2023-11-15

**Authors:** Panagiotis Ioannidis, Dimitrios Katsaras, Theodoros Zografos, Panagiotis Charalambopoulos, Konstantinos Kouvelas, Georgios Tsitsinakis, Ioannis Raitsos-Exarchopoulos, Theodora Kappou, Anastasios Zagoraios, Panagiotis Ganas, Alexandros Vassilopoulos, Emmanouil Xylakis, Evangelia Christoforatou

**Affiliations:** 1 Heart Rhythm Center, IASO Hospital, Athens, Greece

**Keywords:** Box lesion, persistent atrial fibrillation, posterior wall isolation, radiofrequency ablation

## Abstract

The left atrial posterior wall (PW) is known to be a critical substrate for the initiation and perpetuation of atrial fibrillation (AF) and has been explored as a target for catheter ablation, particularly in persistent AF (PerAF). In this retrospective study, we investigate the clinical outcome of patients with PerAF who underwent PW isolation (PWI) restricted in predetermined lines in addition to pulmonary vein isolation (PVI). One hundred consecutive patients (64 ± 9.1 years, 66% male, 20% with previous PVI ablation) underwent PWI in a box lesion setting for PerAF lasting >3 months (34% long-standing PerAF). PW triggers were defined as either foci from the PW that repeatedly induced AF or as isolated AF or atrial tachycardia (AT) within the PW. After a mean follow-up period of 25.6 ± 6.7 months, 61% of the patients remained in sinus rhythm after the last procedure. In 79 patients, the PW was successfully isolated, while, in 21 patients, complete isolation was not possible due to failure in completion of the roof line (n = 16), the floor line (n = 7), or both (n = 2). Patients with incomplete isolation had similar AF/AT recurrence rates compared to those with complete PWI. In 12 patients, PW triggers were identified, and PWI in these patients was shown to have a significantly better prognosis in terms of sinus rhythm maintenance (*P* = .031). Failure of complete PWI does not predispose a patient to an inferior outcome nor is it responsible for iatrogenic ATs. The presence of AF triggers within the PW leads to a particularly favorable result after box lesion isolation.

## Introduction

The left atrial (LA) posterior wall (PW) shares a common embryologic origin with the pulmonary veins (PVs).^1^ Its particular histological and anatomical construction make it an important substrate for the maintenance of atrial fibrillation (AF). It has been reported that the PW is a source of triggers for AF initiation and its electrophysiological (EP) properties promote the perpetuation of AF.^2^ Moreover, it is also known that more prolonged episodes of AF result in remodeling with EP and structural changes that favor the maintenance of AF.^3^ Consequently, PW isolation (PWI) offers a reasonable approach for AF prevention. The existence of AF in patients with isolated PVs probably necessitates additional substrate modification, and certainly the PW is a critical and feasible area for intervention. Indeed, there are a lot of supporting data at the clinical level,^4–6^ although there is also evidence that does not support the necessity of PWI, at least during the first ablation procedure.^7,8^ However, the inability to perform transmural lesions with current tools may hinder the influence of PWI in sinus rhythm (SR) maintenance. Notably, in several series, PWI is mainly achieved with ablation within the PW,^7–9^ apparently because complete PWI is not always possible with ablation at its boundaries.

In this retrospective study, we investigated the clinical outcome of patients with persistent AF (PerAF) of >3 months’ duration who underwent PWI in addition to PV isolation (PVI) in a box lesion setting with radiofrequency (RF) ablation restricted only at the predetermined lines at the roof and floor of the left atrium, then tried to define factors that predispose to recurrence.

## Methods

### Patient population

The study population included 100 consecutive patients who underwent LA PWI in addition to PVI, from January 2020 to January 2022, for PerAF lasting >3 months. Patients who had undergone multiple (>1) previous procedures or procedures with additional ablation beyond PVI, other than cavotricuspid isthmus (CTI) ablation, were excluded from the study. All patients underwent baseline evaluation with clinical history, physical examination, electrocardiography (ECG), and transthoracic echocardiography. The definitions and terminology regarding AF in this article are in agreement with the latest Heart Rhythm Society (HRS)/European Heart Rhythm Association (EHRA)/European Cardiac Arrhythmia Society (ECAS)/Asia Pacific Heart Rhythm Society (APHRS)/Latin American Society of Electrophysiology and Cardiac Stimulation (SOLAECE) Expert Consensus Statement on Catheter and Surgical Ablation of AF.^10^ All patients provided written informed consent. The study protocol was approved by the hospital’s ethics committee and complied with the Declaration of Helsinki.

### Electrophysiological study and catheter ablation

All anti-arrhythmic medications except for amiodarone were discontinued for at least five half-lives before ablation. All patients had the procedure on uninterrupted oral anticoagulation. For patients taking vitamin K antagonists, an international normalized ratio between 2.0 and 3.0 was a prerequisite for the preoperative period as well as for the day of the procedure. For patients taking direct oral anticoagulants with twice-daily dosing, the morning dose was also given. Transesophageal echocardiography was performed in all patients immediately before the beginning of the procedure to exclude LA thrombi. The ablation procedure was performed under general anesthesia.

Twelve-lead ECG and intracardiac electrograms were continuously monitored and stored on a computer-based digital amplifier/recorder system (WorkMate Claris Recording System; Abbott, Chicago, IL, USA). Intracardiac electrograms were filtered from 30–500 Hz. The EnSite Precision three-dimensional (3D) system (Abbott) or the CARTO 3 (Biosense Webster, Diamond Bar, CA, USA) was used for the mapping of the left and right atria if appropriate.

Barium ingestion, before general anesthesia, was used for real-time fluoroscopic imaging of the esophagus. Furthermore, esophageal temperature monitoring was performed with a standard temperature probe (Temperature Cable, 400 Series Probes; GE Medical Systems, Chicago, IL, USA).

A 100-IU/kg dose of heparin was administered immediately after the insertion of the femoral sheaths. Subsequent boluses of heparin were administered every 1 h targeting an activated clotting time of >300 s.

The following diagnostic catheters were introduced via the femoral veins: (1) a quadripolar catheter was positioned in the His-bundle area and (2) a deflectable decapolar 6-French catheter was positioned in the coronary sinus.

A single transseptal puncture was carried out with the aid of an intracardiac echocardiography catheter (ViewFlex Xtra; Abbott) connected to a compatible ultrasound console (Philips CX50; Eindhoven, Netherlands). A 20-pole, variable-radius circumferential mapping catheter (Reflexion Spiral [Abbott] or lasso [Biosense Webster]) or a multipolar high-density mapping catheter (Advisor HD Grid Mapping Catheter [Abbott] or PentaRay^®^ [Biosense Webster]) was introduced through a long transseptal sheath (Preface [Biosense Webster] or LAMP 45° [Abbott]) to guide geometry creation, high-density activation and voltage mapping, and PVI.

A contact force ablation catheter (TactiCath Sensor Enabled™ [Abbott] or ThermoCool SmartTouch^®^ SF [Biosense Webster]) was inserted into the left atrium through a steerable transseptal sheath (Agilis 71 cm [Abbott] or CARTO Vizigo [Biosense Webster]). An anatomical 3D LA model was systematically created by precisely delineating the body of the left atrium with the mapping and ablation catheters.

During the initial AF ablation procedure, a wide antrum PVI was attempted. After placing the 20-pole circumferential PV mapping catheter in each PV or between the ipsilateral PVs, if the anatomy was appropriate, a point-by-point circumferential lesion set encompassing the ipsilateral PVs was created, aiming at complete electrical PV disconnection. RF energy was applied with an upper power of 35 W and a contact force of 10–30 g. At each ablation lesion, a force–time integral (FTI) of >400 gs or an ablation index (AI) of ≥550 at the anterior wall and ≥400 at the PW was sought in the case where EnSite or CARTO 3 was used. If the ablation catheter was in close proximity to the esophagus, which was fluoroscopically visible with barium, the power was reduced to 25 W. Moreover, RF delivery was stopped as soon as the esophageal temperature reached 39°C, even if the ablation target value had not been reached. The energy delivery was not resumed on the PW until the esophageal temperature reached the baseline level. If there was evidence of residual conduction due to inefficient energy delivery, the ablation lesion was delivered inside or outside of the preplanned line, at a safe distance from the esophagus. During the redo procedures, the PV antra were meticulously re-mapped for conduction recurrence and, if such an electrical reconnection was observed, additional lesions were administered, using the aforementioned ablation settings, to achieve PVI.

For the EP evaluation of atrial tachycardias (ATs), local activation time and propagation maps as well as conventional entrainment maneuvers were used to clarify the AT mechanism.

### Posterior wall isolation ablation

**Figure 1:**
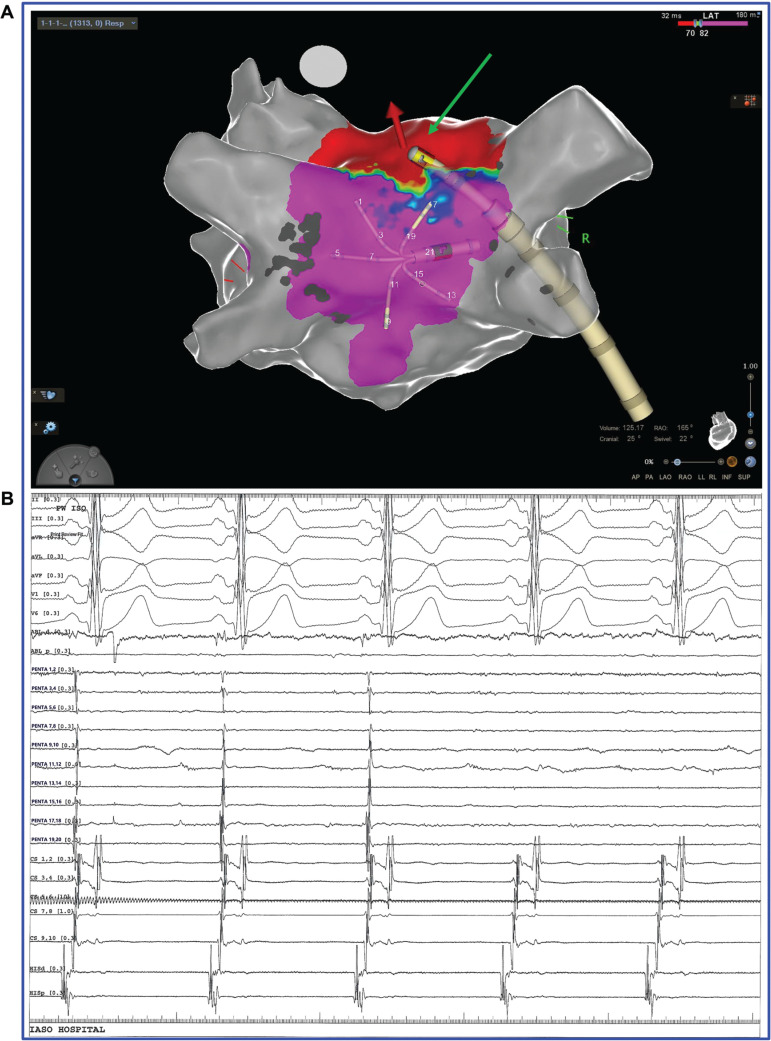
**A:** Detailed mapping with the PentaRay^®^ catheter revealed the gap in the roof line of the left atrium, while the floor line had already been completed **(Video 1)**. **B:** The radiofrequency energy in this area results in complete posterior wall isolation.

The decision to proceed to PWI on top of PVI was at the operator’s discretion, considering factors such as the duration of the arrhythmia, evidence of diseased atrial substrate, or previous PVI. The PWI was started either in AF or in SR after electrical cardioversion according to the operator’s preference. The LA roof line was performed with point-by-point ablation joining the superior border of the circumferential isolation of the contralateral PVs. Similarly, the LA floor line was performed parallel to the roof line, joining the lower border of the contralateral PVs. In each ablation lesion, a contact force of 10–35 g was applied with the ablation catheter supported by the steerable sheath. If the achieved pressure was <10 g, the ablation lesion was not attempted to avoid edema, which could potentially hinder the formation of a transmural lesion. The multipolar mapping catheter was placed in contact with the PW, and the endpoint of PWI was the complete elimination or dissociation of all local electrograms. In the case of conduction maintenance in the PW, after the initial performance of the linear lesions, detailed mapping was performed to highlight the entry points and additional lesions were attempted **(Figure 1 and Video 1)**. If the conduction remained, despite persistent attempts to ablate gaps in roof or floor lines, no further ablation was attempted within the PW. In the case of RF applications in proximity to the esophagus, the precautions described previously were followed.

### Trigger definition and identification

In the present study, we defined an AF trigger as (1) an area exhibiting arrhythmic activity that can spontaneously and repeatedly (≥2 times) initiate AF during the EP study or (2) an isolated arrhythmic activity lasting for >2 s detected within the isolated area of either the PVs or the PW. The identification and mapping of possible AF-initiating triggers were performed after the PVI. Electrical cardioversion was attempted as the catheters were placed in predefined positions to record the AF onset. Specifically, we placed the catheters as follows: (1) the ablation catheter into the LA appendage, (2) the circumferential or the multipolar mapping catheter in the PW, and (3) the quadripolar catheter in the high right atrium in contact with the interatrial septum. Regarding the first criterion, in order to define an area as a trigger, abnormal electrical activity within these areas should induce AF at least twice, so this could not be considered as a random event.

### Follow-up

After ablation, anti-arrhythmic drugs were usually discontinued but could be administered at the discretion of the electrophysiologists for a period of 2–3 months. After the blanking period, the anti-arrhythmic drugs were discontinued as long as there was no AF/AT recurrence. Anticoagulants were continued for the first 3 months and stopped when the CHA_2_DS_2_-VASc score was <1 point for men or <2 points for women. All patients were followed up at 1, 3, 6, and 12 months following the ablation procedure and yearly thereafter with scheduled office visits and ECG and 24-h Holter recordings. Unscheduled office visits with ECG recordings took place when there were symptoms. During the entire postprocedural period, the patients were also followed by their referring physicians. Subsequent information was obtained by phone and/or electronic means. Arrhythmia recurrence was defined as any atrial tachyarrhythmia (AT or AF) episode lasting ≥30 s documented in an ECG or Holter recording or during interrogation of an implantable cardiac rhythm device. A 3-month time interval after PWI ablation was defined as the blanking period.

### Statistical analysis

Continuous variables are summarized as mean ± standard deviation or as median (interquartile range) values according to data normality and were compared using Student’s *t* test or the Mann–Whitney rank-sum test. Categorical data are summarized as frequencies and percentages and were compared using Pearson’s chi-squared test and Fisher’s exact test. Between-group differences were assessed using one-way analysis of variance or the Kruskal–Wallis test, according to data normality. Rates for freedom from arrhythmia recurrence were determined using the Kaplan–Meier analysis and were compared using the log-rank test across groups. To identify independent predictors of arrhythmia recurrence after ablation, we established an appropriate prediction model using logistic regression analysis. Data analyses were performed using IBM SPSS Statistics version 25 (IBM Corporation, Armonk, NY, USA). *P* < .05 was considered statistically significant.

## Results

### Patient characteristics and interventional outcome

The baseline clinical characteristics of the patient cohort are summarized in **Table 1**. All patients had a successful PVI or re-isolation by filling the gaps if PVI had been performed in a previous procedure. The PWI was successful in 79 of 100 patients (79%) at the index procedure. In 21 patients, the PWI was incomplete at the index procedure. The roof line was not completed in 16 patients, the floor line was not completed in 7 patients, and both lines were left incomplete in 2 patients **(Table 2)**. In 20 patients with previous PVI ablation, the evaluation of PV reconnection during the index procedure revealed that 31 of 80 PVs were reconnected and 15 of 20 patients had at least one PV reconnected. In 68 patients, the arrhythmia was terminated with synchronized cardioversion, while 32 patients developed AT during the procedure, which was approached by mapping and targeted ablation. CTI ablation was performed during the index procedure in 30 (30%) patients (in 10 for common atrial flutter and in 20 empirically). A posterior mitral isthmus line was performed in 32 (32%) patients during the index procedure (in 22 due to perimitral atrial flutter and in 10 empirically).

### Arrhythmia recurrences and prognostic factors

**Figure 2:**
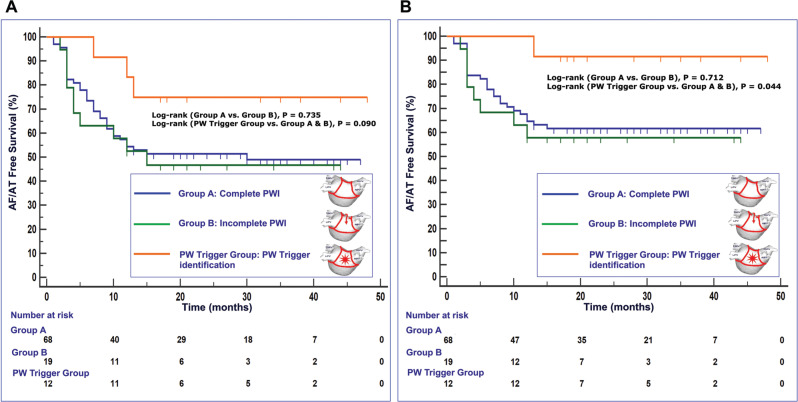
Kaplan–Meier curves of atrial tachycardia/atrial fibrillation–free survival after the index procedure **(A)** and the last procedure **(B)** in (1) group A containing patients with complete PWI without trigger identification, (2) group B containing patients with incomplete PWI without trigger identification, and (3) the PW trigger group containing patients with PW trigger identification who underwent posterior wall isolation. *Abbreviations:* AF, atrial fibrillation; AT, atrial tachycardia; PW, posterior wall; PWI, posterior wall isolation.

**Figure 3:**
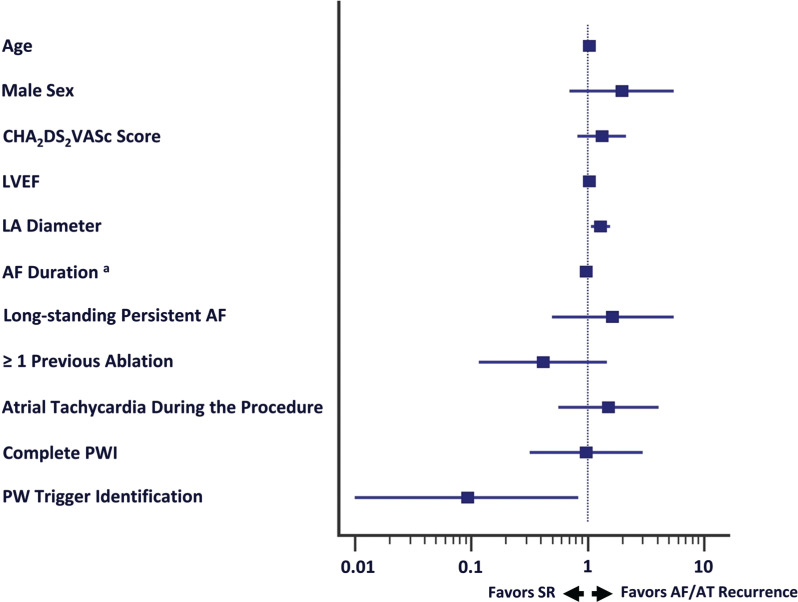
Prognostic factors for atrial tachycardia/atrial fibrillation recurrences. ^a^Time from the onset of persistent atrial fibrillation to the index (box lesion) ablation. *Abbreviations:* AF, atrial fibrillation; AT, atrial tachycardia; LVEF, left ventricular ejection fraction; PerAF, persistent atrial fibrillation; PW, posterior wall; PWI, posterior wall isolation.

After a mean follow-up period of 25.6 ± 6.7 months, 49 patients (49%) were free from atrial tachyarrhythmias after the index procedure, while, after the last procedure, the AF/AT free rate reached 61% **(Table 2)**. Patients in whom the PW was not completely isolated did not differ in long-term prognosis from those in whom the PWI was initially successful (log-rank *P* = .712). Patients in whom an AF trigger was found in the PW had a significantly lower recurrence rate compared to the rest of the patients in the survival analysis (log-rank *P* = .031) **(Figure 2)**. Using logistic regression analysis, the prognostic significance for arrhythmia-free survival was examined for several patient characteristics. Among them, LA diameter and the presence of a PW trigger were associated with a lower recurrence rate (odds ratio [OR], 1.29; 95% confidence interval [CI], 1.06–1.57; *P* = .01 and OR, 0.09; 95% CI, 0.01–0.84; *P* = .034) **(Figure 3)**. Of the 39 patients with arrhythmia recurrence, 33 had AF (18 persistent and 15 paroxysmal) and 6 had AT (3 persistent and 3 paroxysmal).

### Triggers in the posterior wall

**Figure 4:**
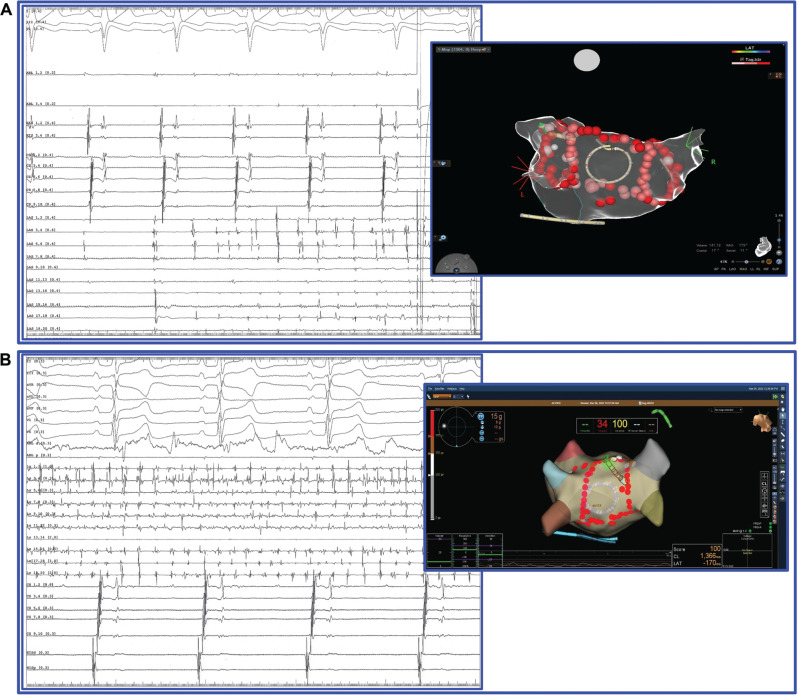
Rapid arrhythmic activity recorded within the isolated PW and SR in the rest atrial myocardium in 2 patients (**A** and **B**). The circumferential catheter is in contact with the posterior wall, and the pulmonary veins are already isolated.

**Figure 5:**
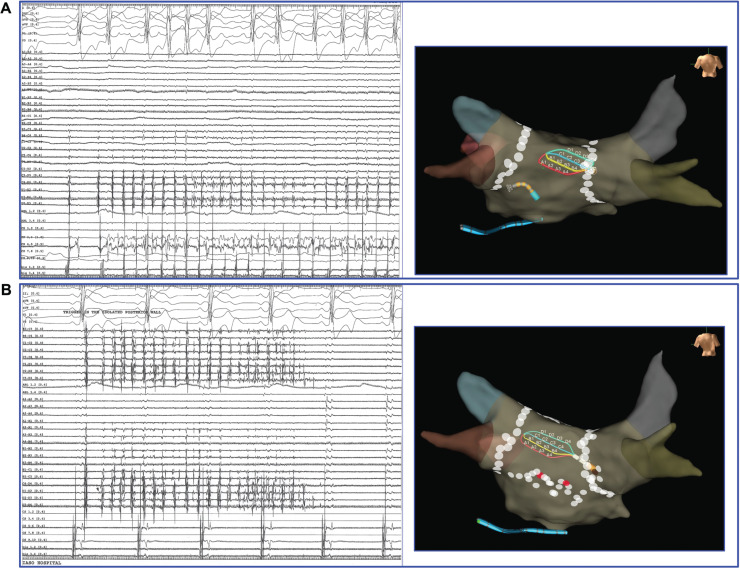
The HD Grid catheter is in contact with the pulmonary wall (three-dimensional images). **A:** Arrhythmic triggering from the pulmonary wall causing initiation of sustained atrial fibrillation after pulmonary vein isolation. **B:** Arrhythmic triggering from the same origin bounded within the pulmonary wall following completion of the box lesion **(Video 2)**. The pulmonary wall trigger this time cannot induce sustained atrial fibrillation, probably due to critical mass reduction.

**Figure 6:**
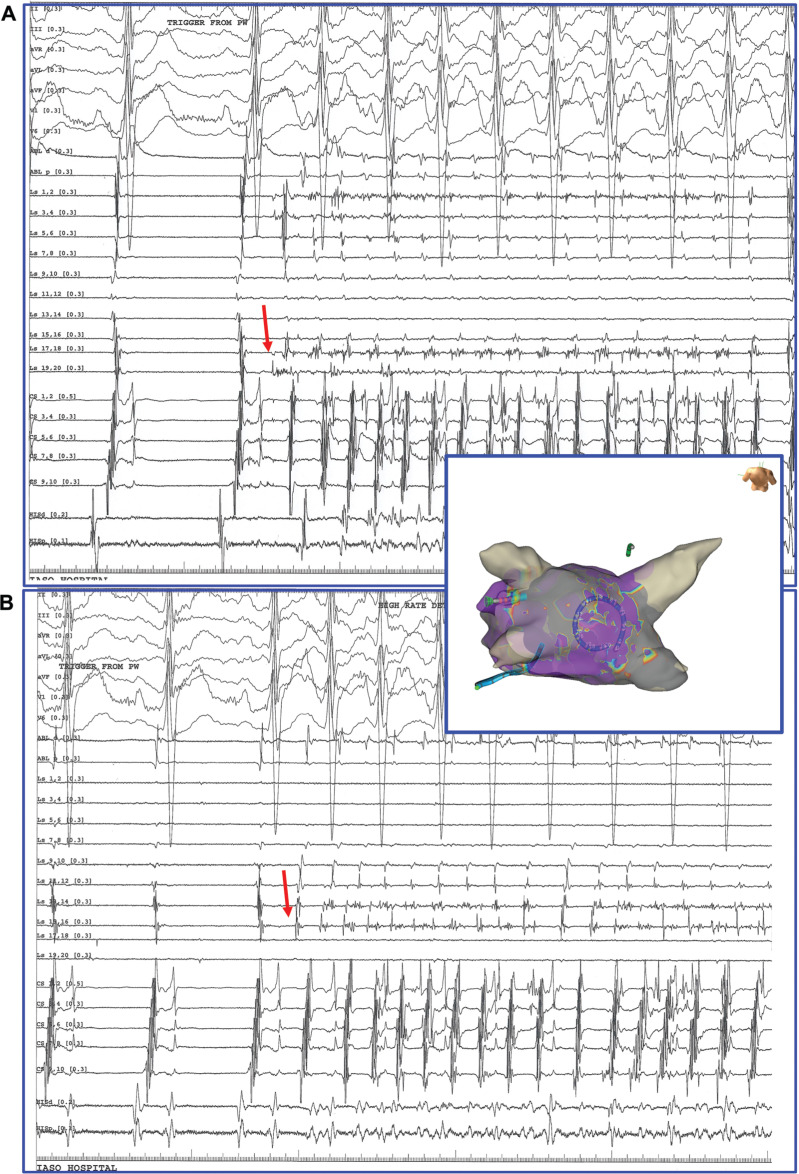
Repeated initiation of atrial fibrillation from the pulmonary wall after pulmonary vein isolation (**A** and **B**). The circumferential catheter is in contact with the pulmonary wall (three-dimensional image) recording the earliest atrial activity (red arrows).

Among the 12 patients in whom a PW trigger was identified, 3 were also found to have isolated arrhythmic activity (AF/AT) in the PW **(Figures 4 and 5) (Video 2)**, while, in the remaining 9 patients, the PW was repeatedly found to be the area of AF initiation **(Figure 6)**. In these patients, complete PWI was achieved except for in one in whom the roof line could not be completed.

### Redo procedures

In 13 of the 100 patients (4 with recurrent AF and 9 with recurrent AT), a redo procedure was performed **(Table 3)**. Among the 11 patients in whom the PW was completely isolated during the index ablation procedure, there was recurrence in only 2 patients (18.2%), including one at the roof line and the other at the floor line. The recurrence rate in the PVs was relatively low, as 5 of 52 PVs (9.6%) were reconnected, and 9 of 13 patients had all PVs isolated. Both lines with conduction recurrence as well as those that were not initially completed were successfully treated with appropriate additional ablation lesions.

### Complications

In our series, the ablation procedure was relatively safe, and only one patient presented with a femoral pseudoaneurysm, which was managed conservatively.

## Discussion

### Main findings

The most important findings of our study are as follows: (1) PWI following a box lesion in patients with PerAF or long-standing PerAF results in long-term maintenance of SR in a significant percentage of patients, with an excellent safety profile; (2) the interventional failure of complete PWI does not appear to have prognostic significance for SR maintenance or increased incidence of iatrogenic ATs; and (3) the identification of AF triggers originating from the PW is a favorable prognostic factor for SR maintenance in patients subjected to PVI plus PWI ablation.

### Interventional and long-term outcomes

The necessity for PWI, at least during the first ablation procedure in patients with PerAF, has not yet been clarified. It is known that, in some patients with PerAF, isolation of the PV antrum is sufficient. However, there is a large proportion of patients who continue to have AF while the PVs are isolated, and it is questionable whether the very limited areas of reconnection often found in redo procedures are responsible for the continuation of AF or whether a more extensive intervention is required.

One of the possible explanations for the beneficial effect of PWI is the hypothesis of critical mass reduction^11^ **(Figure 5B)**. Probably, the remaining substrate is not capable of AF maintenance, and therefore a persistent arrhythmia can be converted to a non-persistent form. Indeed, in our series, which included patients who were in AF for a period of >3 months, 18 of 39 patients with AF/AT recurrence had a paroxysmal atrial tachyarrhythmia, which may be more easily manageable with non-invasive means. Furthermore, the specific lesion setting of PVI and PWI may give the ground to the anterior left atrium for the development of perimitral ATs. In many cases, in this particular substrate, only ATs and not AF can prevail. Therefore, given the fact that ATs can be easily treated with advanced mapping technology, there are significant chances of SR restoration and maintenance.^12^

It is well known that, with conventional catheters and energy sources, it is difficult to create complete and transmural linear lesions in the left atrium.^13^ The amount of energy that should be applied for this purpose is not strictly defined, and therefore, for roof and floor lines, we imply at least the appropriate FTI or AI for PVI. Consequently, the endpoint was the complete elimination of atrial potentials by disconnecting the PW from the rest of the atrial myocardium. This is a very reliable criterion, as, in this area, no far-field potentials of large amplitude are encountered. Conversely, if PWI is performed with ablation within the confines of the PW and relies on diminution of the local electrograms,^7–9^ probably, this may not be a strong indicator of lesion transmurality and thus complete electrical inactivation of the PW. Indeed, the failure to achieve transmural lesions by ablating in an extensive area may create a non-homogenous scar, which, as we know, does not prevent, but, on the contrary, favors AF.^14^ Consequently, the implementation of this technique may have a negative effect on SR maintenance. Interestingly, in previous studies that did not show the superiority of PWI over PVI, the majority of patients required ablation within the “box area.”^7,9^ Instead, the disconnection of PW electrograms by ablating at the predetermined lines is perhaps more confirmatory of PWI by achieving the exemption of the entire area. Within this context, we can notice some similarities of PWI with the evidence we have from wide antral circumferential ablation of PVs. Interestingly, a lower AF recurrence rate has been achieved in patients with bilateral “en bloc” PVI, as compared to those who needed additional carina ablation.^15^

The completeness of the roof line had the higher failure rate, probably because the wall thickness in this area is greater than in the inferior part of the left atrium or the antral region of the PVs.^16^ The epicardial connections of the right atrium with the PW are well-defined anatomical entities^17^ and probably hinder complete PWI. The endocardial ablation may be sufficient for the PVI or the complete elimination of atrial electrograms at the level of roof and floor lines but might fail to ablate these epicardial junctions. In our study, we avoided ablating within the box lesion in cases where there was a resistant line gap or evidence of epicardial connection. In such cases, we could have probably eliminated the endocardial breakthrough of the epicardial pathway. On the contrary, the presence of an epicardial pathway leading blindly to a PW electrically isolated from the rest of the left atrium is unlikely to contribute to the maintenance of AF as, according to the multiple wavelet hypothesis, a slowly conducting gap could result in a reduction in the number of electrical impulses per time unit that pass from the PW to the rest of the atria and vice versa.^18^ Additionally, this setting could also prevent AT, as even one successful line can hinder a roof-dependent atrial flutter. This was probably reflected in the clinical outcome, where there was no significant difference in AT/AF recurrences in patients with complete or incomplete PWI.

Regarding the durability of PWI, we found that, in the cases with initially complete isolation, the PWI persisted in 82% of cases. Furthermore, when PWI was not initially achieved, it became possible in those patients who were submitted to a redo procedure.

### Prognostic factors for atrial fibrillation/atrial tachycardia recurrence

As we found in logistic regression analysis, the only factors significantly associated with arrhythmia recurrence were the absence of PW triggers (discussed in detail below) and the LA dilatation. It is well known that LA enlargement is one of the most important predictors for recurrence after AF ablation.^19^ The greater probability of AF recurrence in patients with larger LAs may, among other reasons, reflect the fact that the atrial mass excess can maintain AF according to the critical mass hypothesis.^11^ Conversely, factors expected to influence prognosis, such as the left ventricular ejection fraction (LVEF), patient age, the CHA_2_DS_2_-VASc score, and the duration and type of AF, did not appear to have a statistically significant prognostic value. This could be explained in part, at least for the type of AF and previous AF ablation, by the high variance of the sample, giving wide CIs. Thus, despite the fact that there was a trend for long-standing persistent type favoring AF and previous AF ablation favoring SR, there was no statistical significance. Moreover, regarding the LVEF, most of the study patients had preserved LVEF. Consequently, LVEF did not seem to adversely affect procedural success because there were no actual subgroups of LVEF for risk stratification.

### The role of triggers in the posterior wall

It is reported that localization and elimination of triggers during AF ablation is beneficial in terms of SR maintenance.^20^ However, there is uncertainty as to which of the arrhythmic activities that are characterized as triggers actually have some significance in the clinical onset of AF or whether this onset can be considered a random event. For example, it is not known whether frequent premature atrial complexes, often found during AF ablation and characterized as AF triggers, can also play the same role in the clinical onset of AF and therefore whether they should be targeted for ablation. Furthermore, mapping the arrhythmic foci that induce atrial tachyarrhythmias is very challenging to do because usually they do not occur repeatedly, and it is also difficult to find their exact location with the frequently used diagnostic catheters.

Taking these into consideration, in our study, we gave a relatively strict definition for triggers, specifying that AF should be induced at least twice from the same origin. The specific setting of the catheters in our study was mainly oriented to highlight triggers originating from the PW. The existence of a PW trigger was found in 12% of the patients, and this finding was also the reason in many cases for proceeding to PWI. Probably, the observed beneficial outcome of PWI in patients with PW triggers may reflect a more targeted effect on AF mechanisms in these patients.

### Limitations

First, this was an observational retrospective study and, consequently, the study population might have been subjected to selection bias. Moreover, the absence of a “PVI-only” control arm does not allow us to draw a conclusion about the additional contribution of PWI with this technique in SR maintenance for patients with PerAF. Furthermore, it is likely that the PWI success rate might have been >79% if ablation had been attempted within the box lesion boundaries in cases where the isolation was not easily achieved with ablation only at the predetermined lines. The PWI was performed in a very systematic way to achieve complete isolation with reliable endpoints. If this predetermined setting was not respected and a kind of homogenization was done, we might have created slow conduction zones and eventually iatrogenic ATs in an attempt to eliminate all the potentials from the PW. Finally, as most of the patients did not have a cardiac implantable electronic device, we might have underestimated the actual failure rate as there could have been asymptomatic recurrences of paroxysmal AT/AF.

## Conclusion

Complete PWI is not always possible with conventional catheters and energy sources, as there is often an inability to perform transmural lesions. Failure to complete both lines is rare, while patients who had incomplete isolation with only one intact line do not appear to have a worse prognosis in terms of SR maintenance or iatrogenic AT occurrence. Finally, in patients with PerAF, preserved left ventricular systolic function, and a minimally dilated left atrium in which triggers from the PW are revealed, it appears that the strategy of PWI with the box lesion setting on top of PVI might result in favorable prognosis, but further randomized prospective studies are needed.

## Figures and Tables

**Video 1:** The propagation of electrical impulse through a roof line gap. The activation mapping was performed with the Pentaray catheter (Biosense Webster) during pacing from the anterior LA.

**Video 2:** The completion of the floor line in the LA restores the SR, but in the PW still exists sustain atrial arrhythmic activity limited within the box lesion area.

**Table 1:** Patient Baseline Characteristics

	Total (n = 100)
Age (years)	64 ± 9.1
Male sex, n (%)	66 (66%)
Arterial hypertension, n (%)	75 (75%)
Diabetes mellitus, n (%)	12 (12%)
Coronary artery disease, n (%)	13 (13%)
Pacemaker or ICD, n (%)	7 (7%)
Left ventricular ejection fraction < 45%, n (%)	15 (15%)
Left atrial diameter (mm)	45.3 ± 3.2
Left ventricular ejection fraction (%)	52.9 ± 6.6
CHA_2_DS_2_-VASc score	2.3 ± 1.27
Previous PVI ablation, n (%)	20 (20%)
Type of AF	
PerAF, n (%)	66 (66%)
Long-standing PerAF, n (%)	34 (34%)

*Abbreviations:* AF, atrial fibrillation; ICD, implantable cardioverter-defibrillator; PerAF, persistent atrial fibrillation; PVI, pulmonary vein isolation.

**Table 2:** Patient Characteristics and Outcomes According to the Interventional Outcome

	Total (n = 100)	Group A Complete PWI Without Trigger Identification (n = 68)	Group B Incomplete PWI Without Trigger Identification (n = 20)	PW Trigger Group^a^ PWI with Trigger Identification (n = 12)	*P* Value
Preprocedural characteristics					
Age (years)	64 ± 9.1	63.9 ± 9.6	63.1 ± 8.1	66.2 ± 8	.440
Male sex, n (%)	66 (66%)	41 (60%)	15 (80%)	9 (75%)	.227
Left atrial diameter (mm)	45.3 ± 3.2	45.5 ± 3.2	45.6 ± 3.3	44.5 ± 1.5	.191
LVEF (%)	52.9 ± 6.6	53 ± 6.9	51.4 ± 7	53.8 ± 5.7	.549
CHA_2_DS_2_-VASc score	2.3 ± 1.27	2.26 ± 1.31	2.25 ± 1.31	2.58 ± 0.86	.628
Previous PVI ablation, n (%)	20 (20%)	14 (20.6%)	4 (20%)	2 (16.7%)	.502
Type of AF (LSPer), n (%)	34 (34%)	23 (33.8%)	7 (35%)	4 (33.3%)	.810
Time in PerAF, months	13.2 ± 14.3	13.2 ± 14.8	13.8 ± 15.3	12.4 ± 9.6	.792
Procedural characteristics					
Incomplete roof line, n (%)	16 (16%)	0	15 (75%)	1 (8.3%)	<.001
Incomplete floor line, n (%)	7 (7%)	0	7 (35%)	0	<.001
Incomplete roof and floor lines, n (%)	2 (2%)	0	2 (10%)	0	.052
AT occurrence during the procedure, n (%)	32 (32%)	22 (32.4%)	6 (30%)	4 (33.3%)	1.0
Postprocedural characteristics and outcome					
Follow-up period (months)	25.6 ± 6.7	25.3 ± 6.4	25.9 ± 7.2	26.9 ± 8.2	.804
AT/AF recurrence, n (%)					
After the index procedure, n (%)	51 (51%)	37 (54%)	11 (55%)	3 (25%)	.181
After the last procedure, n (%)	39 (39%)	29 (42.6%)	9 (45%)	1 (8.3%)	.065
AT or mainly^b^ AT recurrence, n (%)					
After the index procedure, n (%)	15 (15%)	10 (14.7%)	3 (15%)	2 (16.7%)	1.0
After the last procedure, n (%)	6 (6%)	5 (7.4%)	1 (5%)	0	1.0
AF or mainly^b^ AF recurrence, n (%)					
After the index procedure, n (%)	36 (36%)	27 (38.73%)	8 (40%)	1 (8.3%)	.104
After the last procedure, n (%)	33 (33%)	24 (35.3%)	8 (40%)	1 (8.3%)	.137
Recurrence with paroxysmal AF/AT after the last procedure, n (%)	18 (18%)	15 (22.1%)	2 (10%)	1 (8.3%)	.431
Postprocedural anti-arrhythmic medication,^c^ n (%)	10 (10%)	7 (10.3%)	2 (10%)	1 (8.3%)	1.0
Amiodarone, n (%)	4 (4%)	3 (4.4%)	1 (5%)	0	1.0
Other, n (%)	6 (6%)	4 (5.9%)	1 (5%)	1 (8.3%)	.825
Redo procedures after the index (box lesion) ablation, n (%)	13 (13%)	9 (13.2%)	2 (10%)	2 (16.7%)	.812

*Abbreviations:* AF, atrial fibrillation; AT, atrial tachycardia; LSPer, long-standing persistent; PerAF, persistent atrial fibrillation; PVI, pulmonary vein isolation; PW, posterior wall; PWI, posterior wall isolation. ^a^Patients in whom PW triggers were identified regardless of whether PWI was achieved. ^b^More than 50% of the documented atrial tachyarrhythmia burden. ^c^Systemic anti-arrhythmic medication, over a period of >3 months within the follow-up period (excluding the first 3 months of the blanking period).

**Table 3:** Procedural Findings in Redo Procedures

	Total Patients (n = 100)	Group A Complete PWI Without Trigger Identification (n = 68)	Group B Incomplete PWI Without Trigger Identification (n = 20)	PW Trigger Group^a^ PWI with Trigger Identification (n = 12)
Redo procedures, n (%)	13 (13%)	9 (13.2%)	2 (10%)	2 (16.7%)
Clinical tachycardia before redo ablation				
AF, n (%)	4 (30.8%)	4 (44.4%)	0	0
AT, n (%)	9 (69.2%)	5 (55.6%)	2 (100%)	2 (100%)
PV conduction recurrence in redo procedures				
Patients with ≥1 PV reconnected, n (%)	4 (30.8%)	2 (22.2%)	2 (100%)	0
PVs reconnected per total PVs, n/n′ (%)	5/52 (9.6%)	2/36 (5.6%)	3/8 (37.5%)	0/8 (0%)
PW maintained isolated, n (%)	9 (69.2%)	7 (77.8%)	0	2 (100%)
Conduction in roof line, n (%)	3 (23.1%)	1 (11.1%)	2 (100%)	0
Conduction in floor line, n (%)	2 (15.4%)	1 (11.1%)	1 (50%)	0
Conduction in both lines, n (%)	1 (7.7%)	0	1 (50%)	0
Successful re-isolation in roof line (n/n′)	3/3	1/1	2/2	0
Successful re-isolation in floor line (n/n′)	2/2	1/1	1/1	0
Other lines performed				
CTI line, n (%)	4 (30.1%)	3 (33.3%)	0	1 (50%)
Successful	4	3	0	1
Unsuccessful	0	0	0	0
MI line, n (%)	9 (75%)	6 (66.7%)	2 (100%)	1 (50%)
Successful	8	5	2	1
Unsuccessful	1	1	0	0
Evaluation of complete block in previous ablation lines during redo procedure				
CTI line	4	3	0	1
Complete block confirmed	3	3	0	0
Conduction recurrence successfully completed with ablation	1	0	0	1
MI line	3	3	0	0
Complete block confirmed	2	2	0	0
Conduction recurrence successfully completed with ablation	1	1	0	0

*Abbreviations:* AF, atrial fibrillation; AT, atrial tachycardia; CTI, cavotricuspid isthmus; MI, mitral isthmus; PV(s), pulmonary vein(s); PW, posterior wall; PWI, posterior wall isolation. ^a^Patients in whom PW triggers were identified regardless of whether PWI was achieved.
